# Monitoring OTC drug sales for early detection of respiratory infectious disease outbreaks

**DOI:** 10.3389/fpubh.2025.1661753

**Published:** 2025-12-12

**Authors:** Junjie Li, Chen Wu, Kui Liu, Qinbao Lu, Xinyi Wang, Zheyuan Ding, Tianying Fu, Xuefeng Jiang, Haocheng Wu

**Affiliations:** 1 Zhejiang Provincial Center for Disease Control and Prevention, Hangzhou, Zhejiang, China; 2 Pinghu Center for Disease Control and Prevention, Pinghu, China

**Keywords:** symptom monitoring, OTC drug sales, fever respiratory system diagnostic cluster, early detection, early warning

## Abstract

**Background:**

In recent years, over-the-counter (OTC) drug sales have emerged as a novel indicator for symptom monitoring, attracting widespread attention in public health research globally. This study conducted weekly monitoring of five OTC drug categories related to fever respiratory system diagnostic cluster (FRSDC) — antitussive/expectorant drugs, cold medications, antibiotics, pungent and cool exterior-relieving agents, and influenza medicine — in Pinghu City, Zhejiang Province, from 2022 to 2024. Concurrently, weekly FRSDC cases from Pinghu First People’s Hospital were collected.

**Methods:**

Spearman correlation analysis was used to quantify associations between OTC sales and FRSDC cases, while decision tree models evaluated the reliability of OTC data for early prediction of FRSDC trends.

**Results:**

Results showed significant positive correlations between all five OTC drugs and FRSDC cases, with synchronous seasonal peaks in winter and spring (Spearman’s correlation coefficients ranged from 0.36 to 0.80, all *p* value ≤ 0.0001). Even when OTC drug sales preceded FRSDC cases by one or two weeks, strong correlations persisted (Spearman’s correlation coefficients ranged from 0.28 to 0.79, *p* value ≤ 0.0001). Decision tree analysis revealed that combining antitussive/expectorant drugs and influenza medications effectively predicted FRSDC epidemics with 83.33% accuracy (adjusted *p* value < 0.05).

**Conclusion:**

These findings suggest that monitoring OTC drug sales may serve as a useful early warning indicator for FRSDC, potentially aiding public health response and resource planning.

## 1 Introduction

Winter–spring fever with respiratory diseases (FRDs) threaten public health, but traditional surveillance has lags between symptom reporting and diagnosis—making timely monitoring critical for early alerts (1, 2). Pharmaceutical sales monitoring, an innovative approach used since the 1990s, has key gaps: few studies focus on county-level FRDs surveillance via over-the-counter (OTC) drug sales, and no research validates OTC sales as an early warning indicator for fever respiratory system diagnostic cluster (FRSDC) (3). FRSDC, a standardized grouping of cases with both acute infection (body temperature >37.3 °C or ≤36.0 °C, abnormal white blood cell counts or abnormal leukocyte distribution) and respiratory symptoms (e.g., sore throat, cough, dyspnea).

Pinghu City, situated along the East China Sea coast within the Yangtze River Delta’s prime economic zone. It administers six towns and three subdistricts, with a permanent resident population of 697,000 by 2024 (4). The abrupt temperature drops in autumn and winter usher in a peak period for infectious disease incidence (5). In 2022, Coronavirus Disease 2019 (COVID-19) dominated respiratory disease in Pinghu. By 2023, respiratory infections presented a complex landscape of multi-pathogen involvement. The year 2024 saw pertussis and influenza emerge as the primary respiratory ailments in the region (6). But lacking a robust early monitoring system, hindering medical resource allocation (7, 8).

During the early stages of symptom onset or disease progression, patients often seek self-treatment by purchasing medications from pharmacies. This leads to a significant surge in the sales volume of disease-specific symptomatic drugs compared to usual levels, enabling earlier warnings about potential disease outbreaks (9, 10). However, not all infectious diseases are amenable to early warning through pharmaceutical sales monitoring. Generally, it is applicable to respiratory infectious diseases such as influenza, which are characterized by distinct infectious features, a high proportion of mild cases, and widespread public awareness of appropriate medication use (11, 12). The monitored drugs should also be commonly used by the public, affordable, and widely available in the market (12). Additionally, the distribution of monitored pharmacies should be extensive, with attention paid not only to their quantity but also to the population they serve (13).

Fever represents the body’s most common immune response to infection, and the respiratory tract, as an organ system directly connected to the external environment, is a frequent source of infectious fever (14). OTC drugs, which consumers can purchase directly from pharmacies without a doctor’s prescription, are diverse in variety, with clear and broad indications. They are often characterized by high safety profiles, ease of use, and minimal side effects (15). However, the role of OTC drug sales monitoring in predicting the epidemic intensity of FRSD in Pinghu City remains poorly understood. From 2022 to 2024, we continuously collected confirmed cases of FRSDC from Pinghu First People’s Hospital every week, aiming to explore the correlation strength between the sales volume of related OTC drugs and the epidemic trend of FRSDC.

Spearman correlation analysis can effectively assess the strength of association between OTC drug sales and FRSDC, while revealing any monotonic trends between them (16). As a tree-structured machine learning algorithm, the decision tree is commonly used to address classification or regression problems (17). It recursively performs optimal division of input features, gradually splitting the dataset into subsets of increasing purity, and ultimately uses “leaf nodes” to represent decision outcomes and “internal nodes” to represent feature judgment conditions (18). Decision trees play a vital role in public health fields such as disease diagnosis and prediction of infectious disease epidemic characteristics (19, 20). Therefore, this study combined Spearman correlation analysis with decision trees to explore the strength of association between OTC drug sales in Pinghu pharmacies and FRSDC at the First People’s Hospital of Pinghu, and to evaluate the reliability of using OTC drug sales for early monitoring and warning of disease occurrence and epidemics.

## 2 Materials and methods

### 2.1 Geographical information and infectious disease overview of Pinghu City

Pinghu City, in northeast Zhejiang’s Yangtze River Delta, borders Shanghai north and Hangzhou Bay south. A national top 100 county (called “Golden Pinghu”) with developed economy, it covers 557 sq. km (land) and 1,070 sq. km (sea), administering 6 towns and 3 subdistricts. By end-2024, its registered population was 519,000 and permanent resident population 697,000.

In terms of infectious disease trends: Pinghu’s 2022 febrile respiratory diseases were mainly COVID-19 (emerged in March, no severe/fatal cases by Dec) and influenza (high in July, mostly type A; Jiaxing’s influenza-like cases rose then). Pinghu had a respiratory disease surge in winter 2023, with more pediatric outpatient/emergency visits and acute respiratory infections in Dec; China saw influenza as main respiratory disease, plus rhinovirus, *Mycoplasma pneumoniae*, etc. In 2024, influenza was weaker than 2023 (due to warm winter) and onset earlier.

### 2.2 Data collection

Weekly data, encompassing OTC drug sales and FRSDC cases, were collected in alignment with the standard Gregorian calendar week. Each week was defined as the period from Monday to Sunday, and this cycle was consistently applied throughout the entire study period to ensure temporal consistency of the data. From 2022 to 2024, weekly cases of FRSDC that met the criteria of this study were selected from the outpatient, emergency, and inpatient departments of the First People’s Hospital of Pinghu (including admission records, medical course records, and discharge records) through logical calculation or structured keyword extraction. Meanwhile, weekly sales data of OTC drugs related to FRSDC in Pinghu were continuously and systematically collected over the 3 years via the database of pharmacy online sales platforms.

### 2.3 Definition of OTC drugs

OTC drugs are medications that consumers can purchase directly from pharmacies without a doctor’s prescription. This study continuously collected the weekly sales volumes of five OTC drugs closely related to FRSDC in all pharmacies (including independent pharmacies, chain pharmacies and franchise pharmacies) within the jurisdiction of Pinghu City from 2022 to 2024 through the “Yunyaotong Digital-Intelligent Dual Map system of Pinghu Municipal Bureau of Market Supervision and Administration.” These categories include antitussive/expectorant drugs, cold medications, antibiotics, pungent and cool exterior-relieving agents (often used to disperse wind-heat and relieve exterior syndromes), and influenza medications. However, OTC sales data is susceptible to confounding factors, including seasonal and climatic variations, public health awareness, and pharmacy promotional activities.

Antitussive drugs refer to medications that reduce or suppress coughing by inhibiting the cough reflex center or blocking the cough reflex arc. Expectorant drugs are those that promote sputum excretion by thinning mucus, reducing sputum viscosity, or enhancing ciliary movement in the respiratory tract. Cold medications are mostly compound preparations, commonly used to relieve various symptoms caused by the common cold or influenza (e.g., fever, headache, nasal congestion, runny nose, cough, sore throat). Their core role is to alleviate discomfort and improve quality of life; however, they do not directly kill viruses or cure colds, functioning as symptomatic treatments. Antibiotics are a class of chemical substances produced by microorganisms (such as bacteria, fungi, and actinomycetes) or synthesized artificially, which treat infectious diseases by inhibiting or killing the growth and reproduction of pathogenic microorganisms. Pungent and cool exterior-relieving agents are formulations primarily composed of cooling, exterior-releasing herbs (e.g., mint, mulberry leaves, chrysanthemum, and bupleurum). They function to dispel wind-heat, relieve exterior symptoms, and clear internal heat. Influenza medications are drugs used for the prevention and treatment of acute respiratory diseases caused by influenza viruses (e.g., types A, B, and C). Their core role is to inhibit viral replication, relieve symptoms, and reduce the risk of complications.

### 2.4 Diagnostic definition of FRSDC

For inclusion in this study, FRSDC cases were defined as patients who met both of the following two criteria concurrently:

Clinical symptoms of acute infection (patients must present with at least one of the following): ① fever (body temperature >37.3 °C); ② abnormal white blood cell count (either elevated or decreased) or abnormal leukocyte distribution; ③ elevated C-reactive protein (CRP) levels; ④ chills; ⑤ hypothermia (body temperature ≤ 36.0 °C).

Clinical symptoms of respiratory diseases (patients must present with at least one of the following): ① pharyngeal discomfort, dry throat, or sore throat; ② nasal congestion or rhinorrhea; ③ obvious congestion and edema of the nose mucosa, pharynx, or larynx; ④ cough (new onset or exacerbated); ⑤ dyspnea; ⑥ abnormal breath sounds on auscultation (e.g., moist rales, dry rales, wheezing, or dullness); ⑦ chest pain.

### 2.5 Spearman’s correlation and decision tree

Spearman’s correlation, also referred to as Spearman’s rank correlation coefficient, is a non-parametric statistical method. It quantifies the strength and direction of the monotonic relationship between two variables, making it particularly suitable for data that do not conform to a normal distribution.

Decision trees are machine learning models based on a tree-like structure, designed to address both classification and regression tasks. They recursively partition features to iteratively split datasets into subsets with increasing purity, ultimately forming a decision framework resembling a “flowchart.” Each internal node represents a judgment on a specific feature, branches correspond to the outcomes of these judgments, and leaf nodes denote the final decision results.

In this study, Spearman’s correlation analysis was employed to assess the strength of the association between OTC drug sales volumes and FRSDC cases. Additionally, decision trees were used to evaluate the effectiveness of sales data for the five OTC drug categories in classifying the epidemic intensity of FRSDC, as well as their potential for early monitoring and warning. These analyses aim to provide insights and a theoretical basis for the rational allocation of clinical medical resources. All data analyses in this study were conducted using R (version 4.5.0). All statistical tests were two-tailed, with a *p*-value < 0.05 considered statistically significant.

### 2.6 Ethical approval and informed consent

This study was approved by the Ethics Committee of Zhejiang Provincial Center for Disease Control and Prevention and Pinghu Center for Disease Control and Prevention. All FRSDC cases from the First People’s Hospital of Pinghu signed the informed consent form.

## 3 Results

### 3.1 Geographical location of Pinghu City

**Figure 1:**
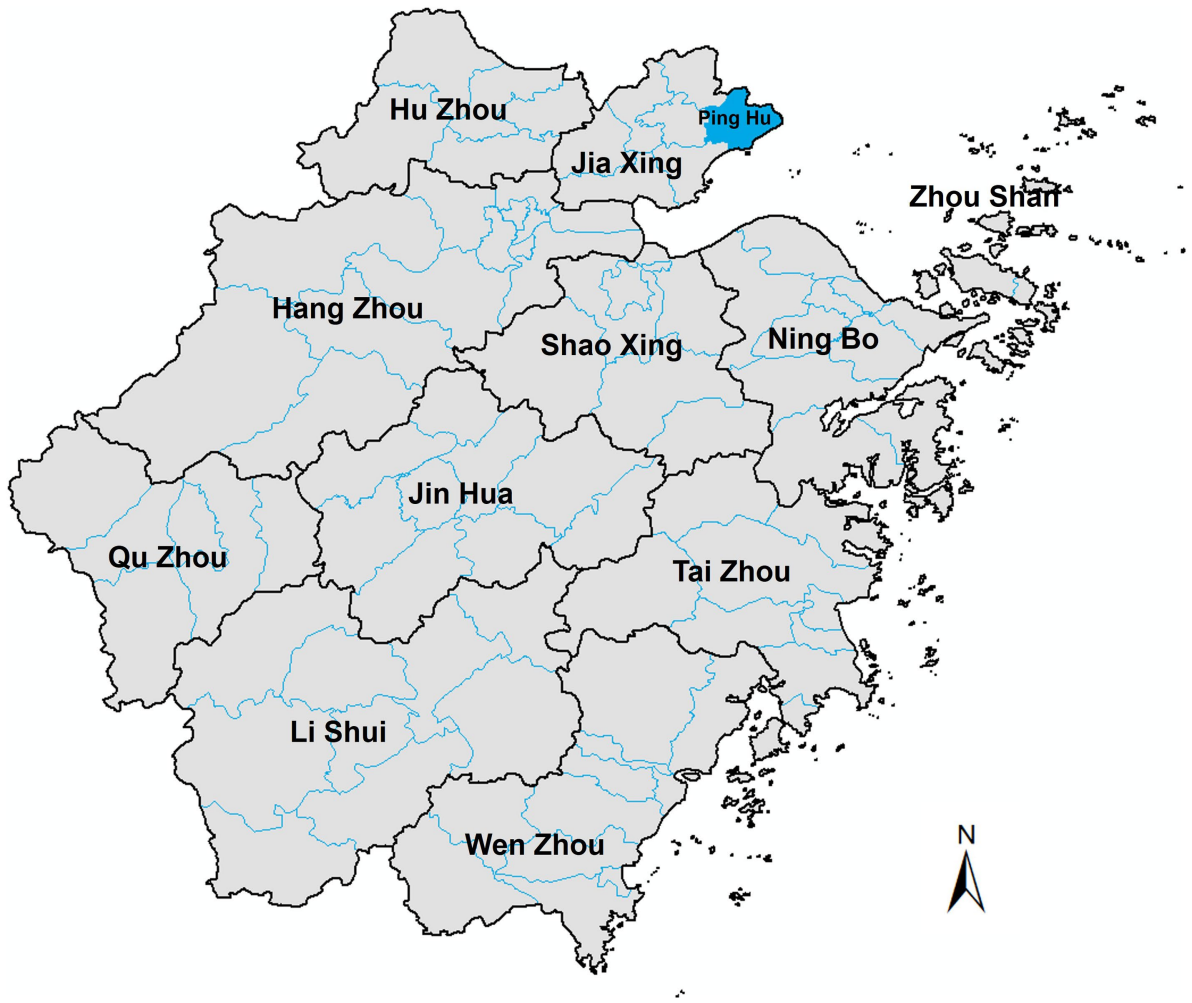
Specific geographical location of Pinghu City, Zhejiang Province. The specific geographical location map of Pinghu City, Zhejiang Province was generated using ArcGIS software.

This study used ArcGIS software to draw the geographical location map of Pinghu City (Figure 1). As shown in Figure 1, Pinghu City is located in the northeastern part of Zhejiang Province.

### 3.2 Correlation between OTC drug sales volumes and FRSDC cases

**Figure 2:**
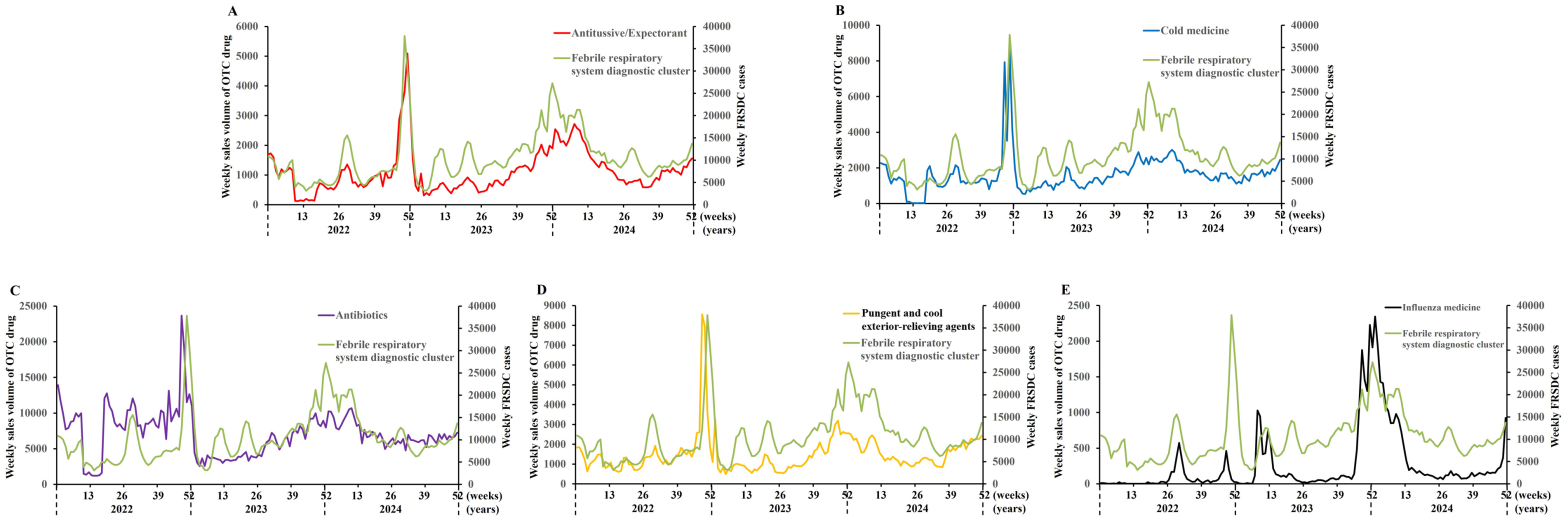
Overall epidemic trends: weekly sales of five OTC drug categories and weekly FRSDC cases (2022–2024). Line graphs were used to, respectively, illustrate the overall trends of weekly sales volumes of the five OTC drug categories—antitussive/expectorant drugs **(A)**, cold medications **(B)**, antibiotics **(C)**, pungent and cool exterior-relieving agents **(D)**, and influenza medications **(E)**—in relation to weekly FRSDC cases from 2022 to 2024.

Spearman correlation analysis was conducted to examine the relationship between the weekly sales volume of five OTC drugs in Pinghu pharmacies and the weekly incidence of FRSDC cases over a three-year period (2022–2024) (Figure 2). The results revealed a significant positive correlation between these two variables. Notably, during the winter and spring seasons of each year, the five OTC drugs and the FRSDC cases exhibited nearly synchronous peak periods (Spearman’s correlation coefficients: 0.36–0.80; *p* ≤ 0.0001; Figure 2; Table 1). Additionally, similar significant positive correlations were observed between FRSDC cases and the sales volume of five OTC drugs one or 2 weeks in advance (Spearman’s correlation coefficients: 0.28–0.79; *p* ≤ 0.0001; Supplementary Figures S1, S2; Table 1).

**Table 1:** Spearman’s correlation between the five OTC drug categories and FRSDC.

OTC drugs	Drugs correspond to symptoms		A week in advance		Two weeks in advance	
	Spearman’s coefficient	p* value	Spearman’s coefficient	p* value	Spearman’s coefficient	p* value
Antitussive/expectorant	0.80	**1.91E−35**	0.74	**5.84E-28**	0.63	**9.74E-18**
Cold medicine	0.79	**3.53E−35**	0.79	**2.29E−33**	0.68	**2.24E−21**
Antibiotics	0.36	**3.00E−06**	0.34	**1.60E−05**	0.28	**6.54E−04**
Pungent and cool exterior-relieving agents	0.66	**7.83E−21**	0.63	**1.63E−18**	0.55	**3.14E−13**
Influenza medicine	0.69	**1.41E−23**	0.74	**2.43E−27**	0.70	**2.15E−23**

All p* values were calculated by Mann–Whitney U test. Significant *p* values were in bold.

### 3.3 Efficacy of combined antitussive/expectorant and influenza medications in identifying FRSDC epidemic intensity

**Figure 3:**
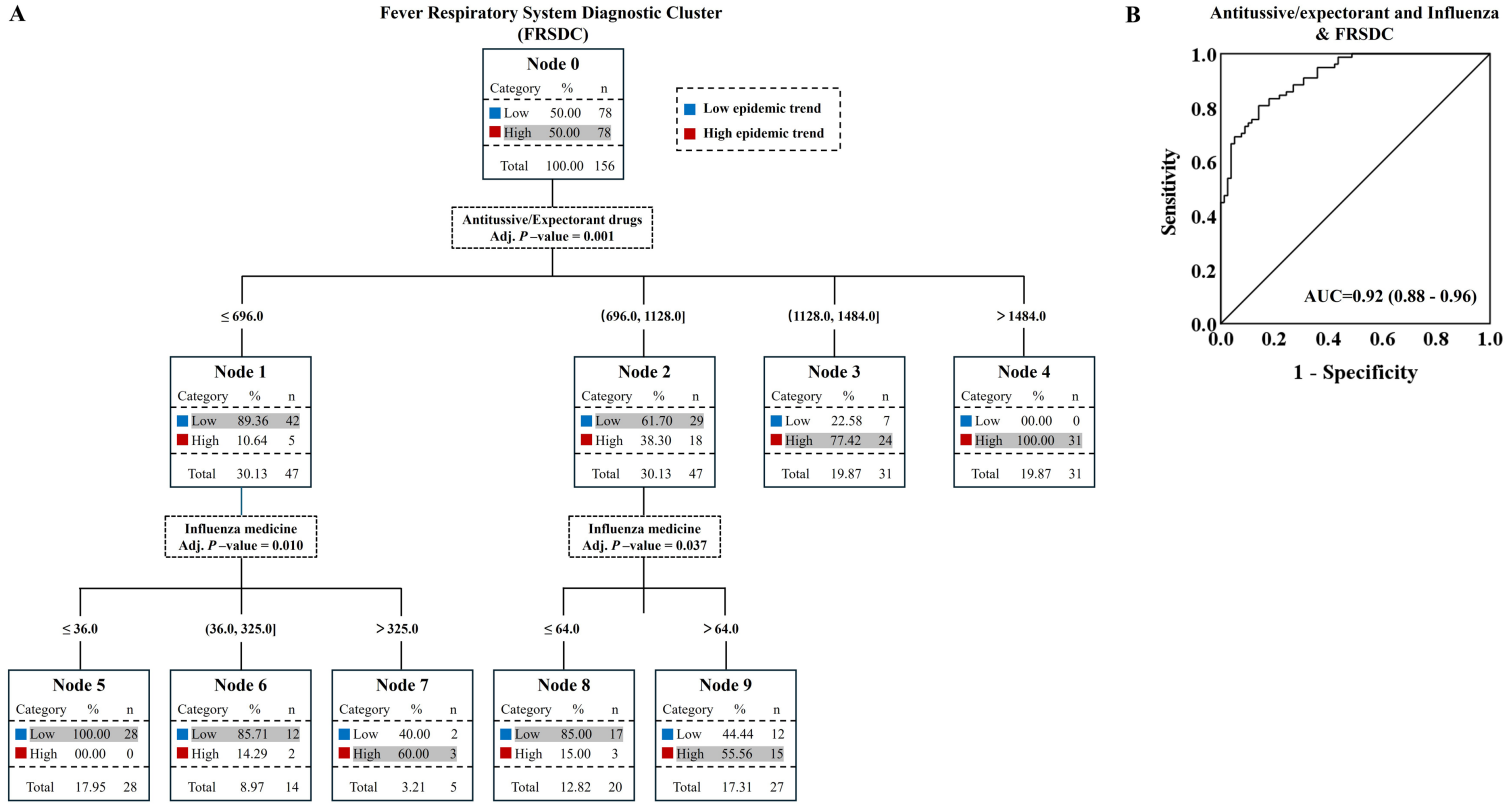
Efficacy of combined antitussive/expectorant drugs and influenza medications in distinguishing FRSDC epidemic intensity**. (A)** The decision tree analysis demonstrated the efficiency of combining antitussive/expectorant drugs and influenza medications in identifying the epidemic intensity of FRSDC cases at the First People’s Hospital of Pinghu. The sales volume of antitussive/expectorant drugs and influenza medications were generated a prediction probability. ROC analysis was conducted on the discriminatory power of the above two OTC drugs in differentiating the epidemic intensity of FRSDC cases **(B)**. The 95% CI of the area under the curve was calculated by logistic regression with adjusted covariates.

In this study, the weekly FRSDC cases from the First People’s Hospital of Pinghu and weekly sales volume of five OTC drugs (antitussive/expectorant drugs, cold medications, antibiotics, pungent and cool exterior-relieving agents, and influenza medications) in Pinghu pharmacies were collected over the period 2022–2024 years (Figure 3). Based on the median value, the 156 weeks of FRSDC cases were divided into two groups: 78 weeks with a high epidemic trend (“High”) and 78 weeks with a low epidemic trend (“Low”). Subsequently, the efficiency of using the above OTC drugs alone or in combination to distinguish between these FRSDC epidemic trends was evaluated. Decision trees partition data into increasingly homogeneous subsets through hierarchical feature-based splitting, essentially learning the “feature-target” mapping rules inherent in the data. These models select optimal thresholds using purity metrics (such as Gini impurity or information entropy) to maximize the homogeneity of resulting child nodes after each split.

Decision tree analysis of this study demonstrated that the combined use of antitussive/expectorant drugs and influenza medications enables relatively efficient differentiation of FRSDC epidemic intensity, with a discrimination efficiency of 83.33% (adjusted *p* < 0.05), indicating statistical significance (Figure 3A; Tables 2, 3). The decision tree constructed in this study comprises 10 nodes (including 7 terminal nodes) with antitussive/expectorant drugs and influenza medications as independent variables. The tree has a depth of 2 layers, with splitting terminated when the number of cases in both parent and child nodes fell below 5 (Supplementary Table S1).

**Table 2:** Decision tree analysis of OTC drugs and FRSDC.

OTC drugs	Single drug	Correct percentage (%)	Two drugs	Correct percentage (%)	Two drugs	Correct percentage (%)
1-Antitussive/expectorant	1	80.81	12	80.82	24	80.12
2-Cold medicine	2	80.12	13	80.83	25	81.41
3-Antibiotics	3	66.71	14	80.80	34	76.32
4-Pungent and cool exterior-relieving agents	4	74.44	**15**	**83.33**	35	73.73
5-Influenza medicine	5	73.72	23	80.11	45	80.14

The bold values represent the most effective OTC drug combination and their discrimination efficiency.

**Table 3:** Decision tree analysis of OTC drugs and FRSDC.

OTC drugs	Three drugs	Correct percentage (%)	Three drugs	Correct percentage (%)	Four drugs	Correct percentage (%)	All drugs	Correct percentage (%)
1-Antitussive/expectorant	123	80.81	145	83.32	1,234	80.82	12,345	83.33
2-Cold medicine	124	80.83	234	80.14	1,235	83.31		
3-Antibiotics	125	83.31	235	81.41	1,345	83.30		
4-Pungent and cool exterior-relieving agents	134	80.82	245	81.42	1,245	83.32		
5-Influenza medicine	135	83.30	345	80.13	2,345	81.42		

To assess the potential clinical application of antitussive/expectorant drugs and influenza medications for differentiating the epidemic intensity of FRSDC cases, receiver operating characteristic (ROC) curve analysis and binary logistic regression were performed. The area under the ROC curve (AUC) was 0.92 with a 95% confidence interval (CI) of 0.88–0.96. Additionally, the sensitivity and specificity were 0.81 and 0.86, respectively (*p* < 0.0001) (Figure 3B).

## 4 Discussion

From 2022 to 2024, this study collected weekly sales data for five OTC drugs across pharmacies in Pinghu, alongside weekly confirmed cases of FRSDC at Pinghu First People’s Hospital. Spearman correlation analysis revealed the sales volume of five OTC drugs in Pinghu City is closely positively correlated with the cases of FRSDC, and the epidemic peaks of both occur almost simultaneously in winter and spring every year. It is worth noting that the above-mentioned correlation still exists between FRSDC cases and OTC drug sales one or 2 weeks in advance. The decision tree further shows that the combined application of antitussive/expectorant drugs and influenza drugs may predict the epidemic intensity of FRSDC.

In recent years, sudden infectious diseases have frequently emerged and spread in China (21). Notable examples include the 2003 outbreak of Severe Acute Respiratory Syndrome (SARS), the first reported case of human infection with highly pathogenic avian influenza (H7N9) in 2013, and the late 2019 outbreak of Coronavirus Disease 2019 (COVID-19) (22, 23). These events have severely threatened human health, economic development, and national security, underscoring the urgent need for timely and sensitive monitoring indicators to enable early prediction and warning of infectious diseases.

Respiratory diseases caused by various pathogens tend to surge during winter and spring, often triggered by abrupt temperature drops (24). For respiratory infectious diseases where the public has basic knowledge of drug treatments, most patients with early symptoms (e.g., fever, cough, runny nose, sore throat) first seek symptomatic relief by purchasing medications at pharmacies (25). Only when symptoms are complex or persistent do patients typically visit hospitals for diagnosis and treatment to avoid delayed care (26). Common OTC drugs used to manage respiratory diseases include antitussive/expectorant drugs, cold medications, antibiotics, pungent and cool exterior-relieving agents, and influenza medications (27, 28). These drugs effectively alleviate mild to moderate symptoms such as colds, coughs, nasal congestion, and sore throats, with well-defined indications and high safety profiles (29). Consequently, during outbreaks of FRDs, sales of these related OTC drugs often rise significantly (10).

FRDs are primarily characterized by symptoms such as fever (body temperature ≥37.3 °C), cough, nasal congestion, and runny nose (30, 31). Traditional monitoring of FRDs relies on data from outpatient, emergency, and inpatient departments of sentinel hospitals. However, such data often lags behind the actual onset of symptoms in the population, resulting in a lack of timeliness (32). In contrast, OTC drug sales monitoring involves systematically and continuously collecting and analyzing the association between drug sales volumes and disease incidence to predict future epidemic trends (10, 33). As an emerging symptom monitoring approach in recent years, OTC drug sales monitoring can enable early prediction and warning of disease outbreaks *prior to* the availability of confirmed hospital case data. This advantage has garnered widespread attention and research interest among public health scholars (11, 34, 35).

Foreign studies have documented links between OTC drug sales and disease outbreaks. For instance, during the 1993 waterborne cryptosporidiosis outbreak in the United States, antidiarrheal OTC drugs in local pharmacies were sold out (36). In 1996, New York adopted OTC drug sales as a monitoring indicator for waterborne diseases (37). Domestically, similar patterns emerged during the initial phase of the COVID-19 epidemic in late 2019, when antiviral drugs (oseltamivir, ribavirin, acyclovir), antipyretic-analgesic drugs (ibuprofen, paracetamol), antibiotics (amoxicillin, cephalosporins), and medical supplies (masks, alcohol, disinfectants) faced widespread shortages (38–40). Numerous studies have confirmed a significant positive correlation between OTC drug sales volumes and the number of hospital-confirmed cases, supporting the use of OTC sales data as an early warning indicator for sudden infectious diseases (41–43). However, research on leveraging drug or medical supply sales monitoring for early warning of disease outbreaks remains limited in China. Against this backdrop, the present study focuses on the role of OTC drug sales in the early monitoring and warning of FRSDC.

The sales volume of OTC drugs is closely related to the epidemic trend of infectious diseases. In the early stage of an infectious disease outbreak, people who show early symptoms will purchase the corresponding OTC drugs, causing the sales of specific types of OTC drugs to change first. A continuous multiple increase in sales for 1–2 weeks often indicates an increase in the number of infections, and the month-on-month growth rate may also coincide with the peak of subsequent confirmed cases. OTC drug sales monitoring involves the continuous, systematic collection of data via pharmacy platform databases, offering advantages such as large sample sizes, ease of access, and straightforward quality control (44).

However, this approach has inherent limitations: it focuses solely on drug sales information, lacks specificity, and relies on a single data source (45). Additionally, OTC sales data are susceptible to confounding factors, including drug inventory levels, pre-holiday/weekend stockpiling, seasonal and climatic variations, regional economic conditions, healthcare insurance policies, public health awareness, age-related differences in medication use (e.g., between children and adults), and pharmacy promotional activities (46). To address potential biases, in addition to tracking individual drug sales volumes, it is necessary to collect total drug sales data and calculate the proportion of individual drugs in total sales. This helps exclude the possibility that increased sales are driven by population growth in the pharmacy’s service area (27). Looking ahead, OTC drug sales monitoring should be integrated with other symptom-based surveillance data—such as school and factory absence records, online and hospital disease-related search queries, and prescription drug sales in medical institutions—to enhance early monitoring and warning of infectious diseases. In addition, the Spearman correlation is non-causal, and decision tree models often have limitations such as easy overfitting, sensitivity to data, and insufficient stability.

## 5 Conclusion

This study found that the sales volume of five OTC drugs in Pinghu was closely and positively correlated with FRSDC cases, and their epidemic peaks almost occurred simultaneously in winter and spring every year. Decision tree analysis could suggest that the sales volume of antitussive/expectorant and influenza drugs may serve as useful indicators for monitoring the epidemic trend of FRSDC disease.

## Data Availability

The datasets presented in this study can be found in online repositories. The names of the repository/repositories and accession number (s) can be found in the article/Supplementary material.

## Supplementary material

Supplementary Figure S1 Overall epidemic trends: weekly sales of five OTC drug categories (1 week in advance) and weekly FRSDC Cases (2022–2024). Line graphs were used to illustrate the overall trends of weekly sales volumes of the five OTC drug categories—antitussive/expectorant drugs **(A)**, cold medications **(B)**, antibiotics **(C)**, pungent and cool exterior-relieving agents **(D)**, and influenza medications **(E)**—1 week in advance, in relation to weekly FRSDC cases from 2022 to 2024.

Supplementary Figure S2 Overall epidemic trends: weekly sales of five OTC drug categories (2 weeks in advance) and weekly FRSDC cases (2022–2024). Line graphs were used to illustrate the overall trends of weekly sales volumes of the five OTC drug categories—antitussive/expectorant drugs **(A)**, cold medications **(B)**, antibiotics **(C)**, pungent and cool exterior-relieving agents **(D)**, and influenza medications **(E)**—2 weeks in advance, in relation to weekly FRSDC cases from 2022 to 2024.
