# Supplementary figures and images for: Monitoring OTC drug sales for early detection of respiratory infectious disease outbreaks
Source: Front Public Health. 2025 Dec 12;13:1661753. doi: 10.3389/fpubh.2025.1661753 (PMC12741107; doi:10.3389/fpubh.2025.1661753)

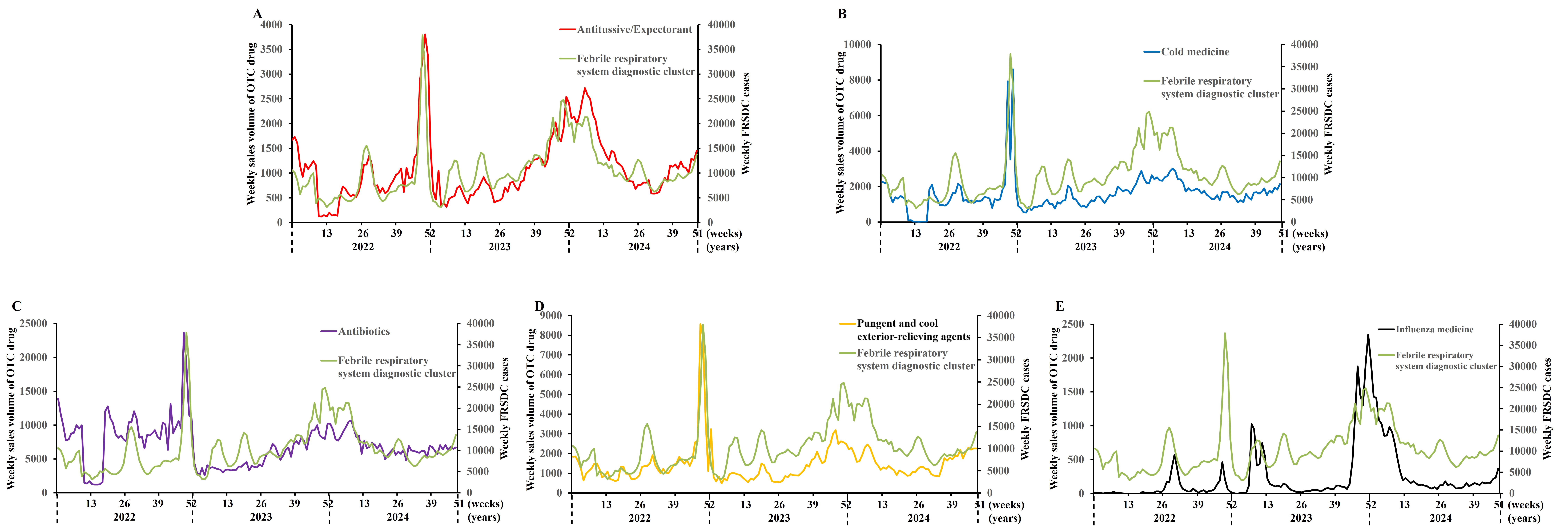

Supplement: Supplementary Figure S1 — Overall epidemic trends: weekly sales of five OTC drug categories (1 week in advance) and weekly FRSDC Cases (2022–2024). Line graphs were used to illustrate the overall trends of weekly sales volumes of the five OTC drug categories—antitussive/expectorant drugs (A), cold medications (B), antibiotics (C), pungent and cool exterior-relieving agents (D), and influenza medications (E)—1 week in advance, in relation to weekly FRSDC cases from 2022 to 2024. [file Image_1.jpeg]

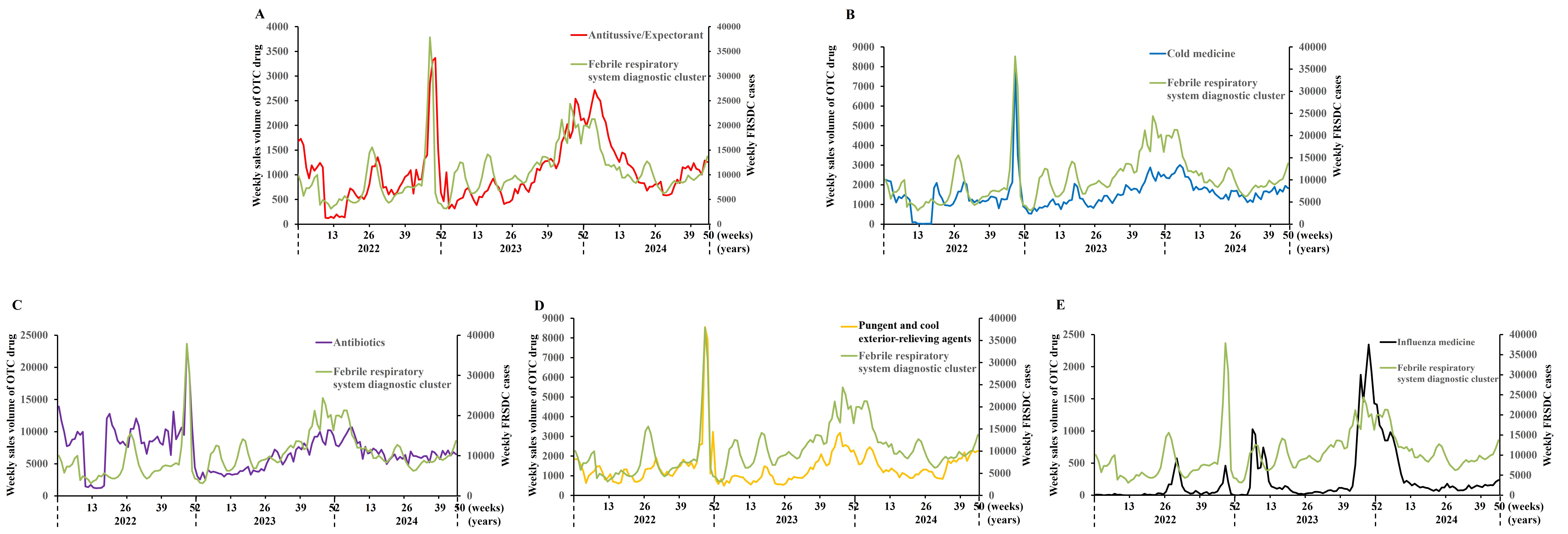

Supplement: Supplementary Figure S2 — Overall epidemic trends: weekly sales of five OTC drug categories (2 weeks in advance) and weekly FRSDC cases (2022–2024). Line graphs were used to illustrate the overall trends of weekly sales volumes of the five OTC drug categories—antitussive/expectorant drugs (A), cold medications (B), antibiotics (C), pungent and cool exterior-relieving agents (D), and influenza medications (E)—2 weeks in advance, in relation to weekly FRSDC cases from 2022 to 2024. [file Image_2.jpeg]
