# Supplementary material for: Monitoring OTC drug sales for early detection of respiratory infectious disease outbreaks
Source: Front Public Health. 2025 Dec 12;13:1661753. doi: 10.3389/fpubh.2025.1661753 (PMC12741107; doi:10.3389/fpubh.2025.1661753)
Supplement: Supplementary file 3 [file Table_1.docx]

**Table S1. Decision Tree Summary**

| **Results** | Independent Variables | Antitussive/ Expectorant drug  Influenza medicine |
| --- | --- | --- |
|  | Minimum Cases in Parent Node | 5 |
|  | Minimum Cases in Child Node | 5 |
|  | Number of Nodes | 10 |
|  | Number of Terminal Nodes | 7 |
|  | Tree Depth | 2 |
| **Classification** | Overall Percentage | 83.33% |
| **Risk** | Std. Error | 0.030 |
